# Effect of treatment with conditioned media derived from C2C12 myotube on adipogenesis and lipolysis in 3T3-L1 adipocytes

**DOI:** 10.1371/journal.pone.0237095

**Published:** 2020-08-05

**Authors:** Kotaro Tamura, Naoko Goto-Inoue, Kaede Miyata, Yasuro Furuichi, Nobuharu L. Fujii, Yasuko Manabe

**Affiliations:** 1 Department of Health Promotion Sciences, Graduate School of Human Health Sciences, Tokyo Metropolitan University, Hachioji, Tokyo, Japan; 2 Department of Marine Science and Resources, College of Bioresource Sciences, Nihon University, Fujisawa, Kanagawa, Japan; University of Minnesota Medical School, UNITED STATES

## Abstract

Regular exercise is an effective strategy that is used to prevent and treat obesity as well as type 2 diabetes. Exercise-induced myokine secretion is considered a mechanism that coordinates communication between muscles and other organs. In order to examine the possibility of novel communications from muscle to adipose tissue mediated by myokines, we treated 3T3-L1 adipocytes with C2C12 myotube electrical pulse stimulation-conditioned media (EPS-CM), using a C2C12 myotube contraction system stimulated by an electrical pulse. Continuous treatment with myotube EPS-CM promoted adipogenesis of 3T3-L1 pre-adipocytes via the upregulation of the peroxisome proliferator-activated receptor-gamma (PPARγ) 2 and PPARγ-regulated gene expression. Furthermore, our results revealed that myotube EPS-CM induces lipolysis and secretion of adiponectin in mature adipocytes. EPS-CM obtained from a C2C12 myoblast culture did not induce such changes in these genes, suggesting that contraction-induced myokine(s) secretion occurs particularly in differentiated myotubes. Thus, contraction-induced secretion of myokine(s) promotes adipogenesis and lipid metabolism in 3T3-L1 adipocytes. These findings suggest the possibility that skeletal muscle communicates to adipose tissues during exercise, probably by the intermediary of unidentified myokines.

## Introduction

Regular exercise, one of the most effective ways to promote physical and metabolic health, is associated with longevity [1]. Although there are multiple mechanisms by which exercise promotes health, the secretion of myokines, cytokines, and peptides by skeletal muscle has also been recognized as a mechanism that coordinates communication between muscles and other organs [2]. Myokines coordinate whole-body energy metabolism by communicating between local and distant organs via autocrine, paracrine, and endocrine systems [3]. Therefore, a better understanding of how exercise-induced myokines exert health benefits may identify novel therapeutic approaches for the promotion of public health.

Exercise prevents obesity and type 2 diabetes partially through adaptations to adipose tissue such as decreased adipocyte size and lipid content, altered expression of adipokines, and "beiging" [4]. As some of the beneficial effects to adipose tissue were expected to be derived from muscle to adipose tissue communication mediated by myokines, several studies have examined the endocrine effects exerted by myokines on adipose tissue function. For example, interleukin 6 (IL-6), one of the most well-investigated myokines [5], increased lipolytic effects on mature 3T3-L1 adipocytes and human visceral adipose tissue [6–8]. Moreover, skeletal muscle-specific overexpression of IL-15 inhibits adipose tissue deposition [9]. Irisin [10], Meteorin-like [11], and β-aminoisobutyric acid (BAIBA) [12] are present in the culture media of muscle cells overexpressing PGC1-α, a transcriptional co-activator that activates various muscle adaptations induced by exercise, and promotes browning and thermogenesis ("beiging") in white adipose tissue. Thus, certain myokines may induce endocrine effects and alter adipocyte metabolism.

Although exercise increases the concentrations of certain myokines in circulating plasma, it remains unclear whether most myokines are secreted by muscle cells or other cells surrounding skeletal muscle (e.g., vessel, neuronal, or blood cells). Thus, *in vitro* models that mimic muscle contraction may be suitable for not only studying signaling events and metabolic adaptations in contracting muscles but also for identifying novel myokines and understanding their secretory mechanisms [13]. Our group previously developed an acute muscle contraction system using electric pulse-stimulated C2C12 myotubes [14]. Using this system, we identified certain novel contraction-induced myokines [15, 16], and candidate myokines (unpublished) in electrical pulse stimulation-conditioned media (EPS-CM).

The current study explored unidentified communication between muscle–adipose tissues by treating 3T3-L1 adipocytes with C2C12 myotube EPS-CM, which is expected to contain numerous contraction-induced myokines. Here, we demonstrate that myotube EPS-CM promotes adipogenesis in 3T3-L1 pre-adipocytes and induces lipolysis in mature adipocytes. Our data showed the possibility of novel muscle–adipose tissue crosstalk via contraction-induced myokines.

## Materials and methods

### Cell culture

C2C12 myoblasts (American Type Culture Collection, Manassas, VA) were seeded onto 4-well rectangular plates, at a density of 2.5×10^5^ cells/well, with 3 mL growth medium, which consisted of Dulbecco's Modified Eagle's Medium (DMEM; 25 mM glucose; Invitrogen, Carlsbad, CA) supplemented with 10% fetal bovine serum (FBS; BioWest, Nuaillé, France) and 1% penicillin-streptomycin (PS; Invitrogen). The cells were maintained in an incubator under the following conditions: 37 °C and 5% CO_2_. Upon reaching 80% confluence, the medium was switched to a differentiation medium, consisting of DMEM supplemented with 2% calf serum (BioWest), 1% non-essential amino acids (Invitrogen), and 1% PS (day 0). The cells were used for experiments on day 5 post-differentiation.

3T3-L1 pre-adipocytes (ATCC, VA, USA) were cultured in DMEM containing 10% FBS and 1% PS. Differentiation was induced on day 2 following confluence by replacing the medium with DMEM containing 10 μg/mL insulin, 2.5 μM dexamethasone, and 0.5 mM 3-isobutyl-1-methylxanthine (AdipoInducer Reagent; TaKaRa, Shiga, Japan) (day 0). Two days later, the medium was replaced with DMEM containing 10% FBS, 10 μg/mL insulin, and 1% PS and changed every two days thereafter. The cells were used for experiments at 4–10 days post-differentiation.

### Cell contraction system and preparation of C2C12 myotube EPS-CM

C2C12 myotube contraction was induced as described previously [14] with slight modifications. Briefly, differentiated C2C12 myotubes were washed twice with phosphate-buffered saline (PBS) and incubated with 2 mL serum-free DMEM (5.5 mM glucose) without phenol red supplemented with 4.0 mM l-glutamine 2 h before the contraction study. The 4-well plates were connected to an electrical stimulation apparatus C-Dish (Ion Optix Corp., MA, USA) and subjected to electric pulse stimulation (EPS) using an electrical pulse generator (Uchida Denshi, Hachioji, Japan). During the experiments, myotubes were incubated with new serum-free DMEM and stimulated with electric pulses as follows: 35 V, 15–20 mA at 1 Hz for 25 ms at 975 ms intervals for 3 h.

**Fig 1:**
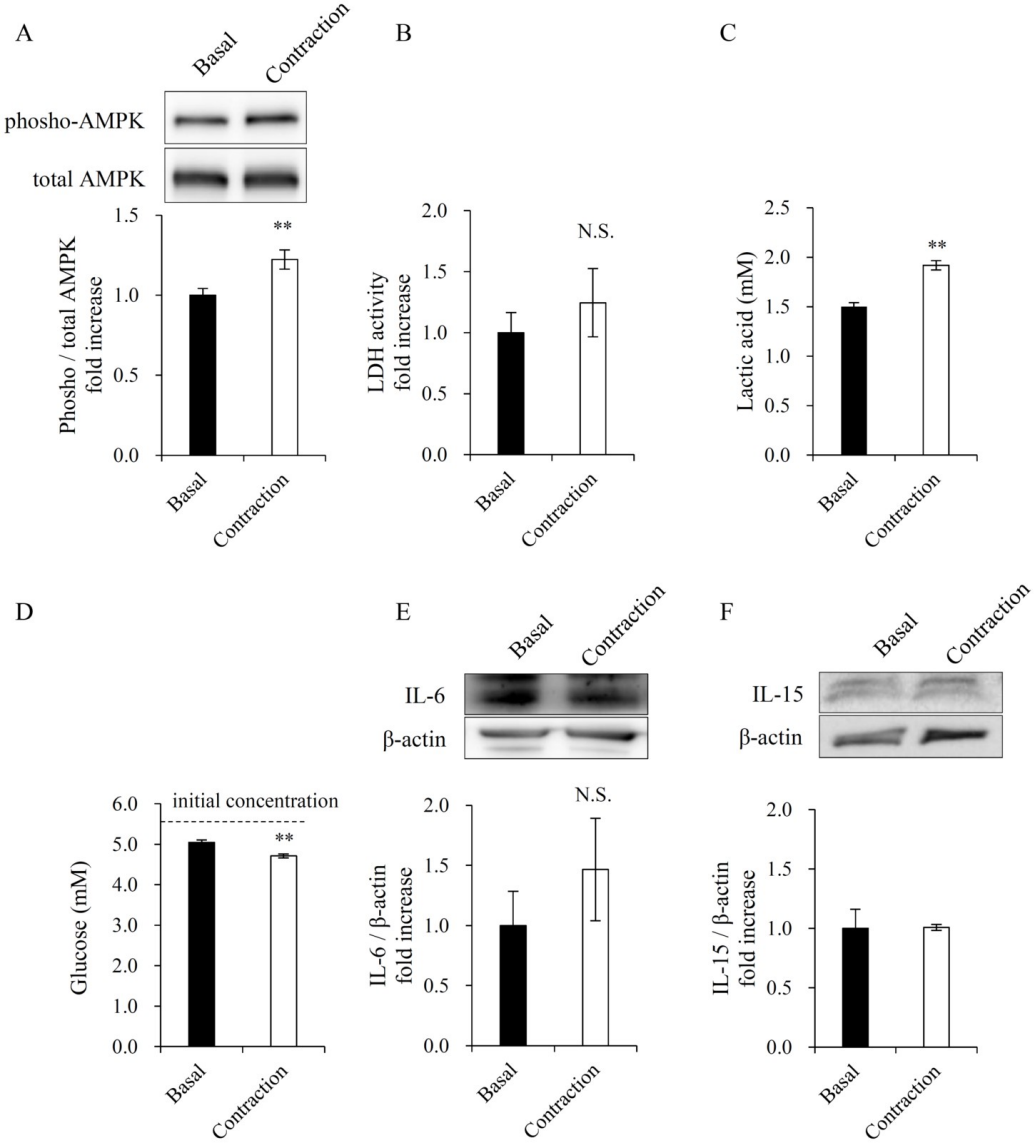
Cell contraction and preparation of C2C12 myotube electrical pulse stimulation-conditioned media (EPS-CM). (A) AMPK phosphorylation (Thr172) level in C2C12 myotubes by 3h EPS. (B) Lactate dehydrogenase activity in the conditioned media. (C, D) Lactic acid and glucose concentration in the conditioned media. The initial concentration of glucose in the medium was 5.5 mM. (E, F) Immunoblotting of IL-6 and IL-15 in the conditioned media. Data are expressed as mean ± SEM (n = 8 (A-E), n = 4 (F)). **; p < 0.01. N.S., not significant.

Following EPS, conditioned media were collected and centrifuged at 12,000 × *g* for 35 min at 4 °C to remove resultant cells. In order to analyze cell damage caused by EPS, lactate dehydrogenase (LDH) activity was measured in the conditioned medium using an LDH assay kit following the manufacturer's instructions (Roche, Basel, Switzerland). The conditioned media were divided into fractions ≥3 and <3 kDa using 3-kDa cut-off centrifugal filters (Millipore, Watford, UK). The ≥3 kDa fraction was subjected to immunoblotting to detect IL-6 and IL-15 (see Immunoblotting section). The lactate acid and glucose content in the <3 kDa fraction was measured using a lactate assay kit and a glucose assay kit, respectively, following the manufacturer's instructions (Dojindo, Kumamoto, Japan). Myotubes stimulated by electrical pulse, and the conditioned media (EPS-CM) were defined as 'Contraction,' and their control (i.e., without EPS) was defined as 'Basal' (Fig 1 and S1 Fig).

### Treatment of 3T3-L1 adipocytes with C2C12 myotube/myoblast EPS-CM

**Fig 2:**
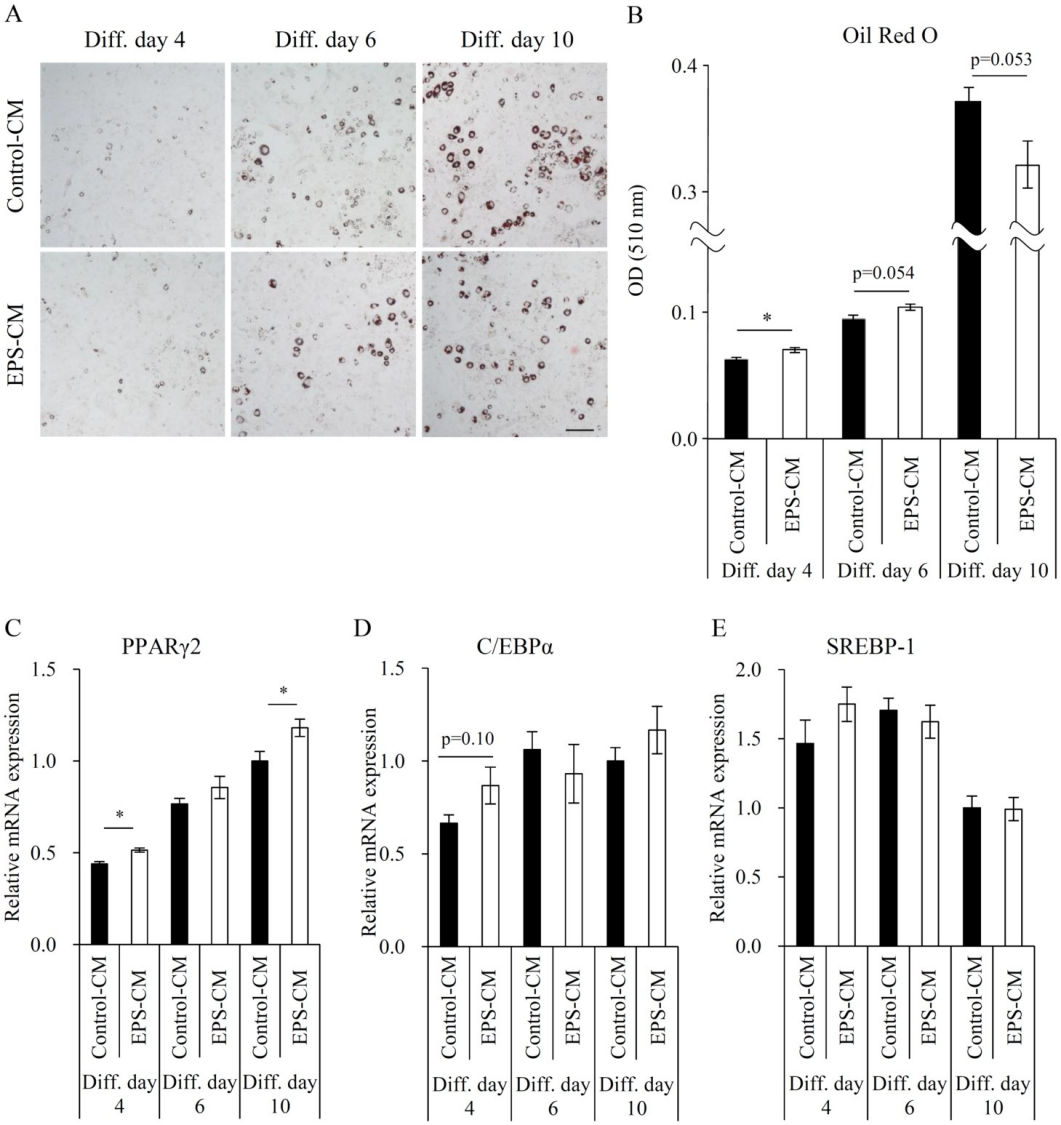
Effects of myotube EPS-CM treatment on differentiation and lipid accumulation of 3T3-L1 adipocytes. Differentiating 3T3-L1 adipocytes were continuously treated with 50% myotube EPS-CM. (A) Representative images of adipocytes stained with Oil Red O on days 4, 6, and 10 post-differentiation, (B) followed by the measuring of total lipid amount. (C-E) The expression levels of PPARγ2, C/EBPα, and SREBP-1 were evaluated via qRT-PCR on days 4, 6, and 10. Data are expressed as mean ± SEM (n = 3–4). *; p < 0.05. N.S., not significant. Scale bar, 200 μm.

**Fig 3:**
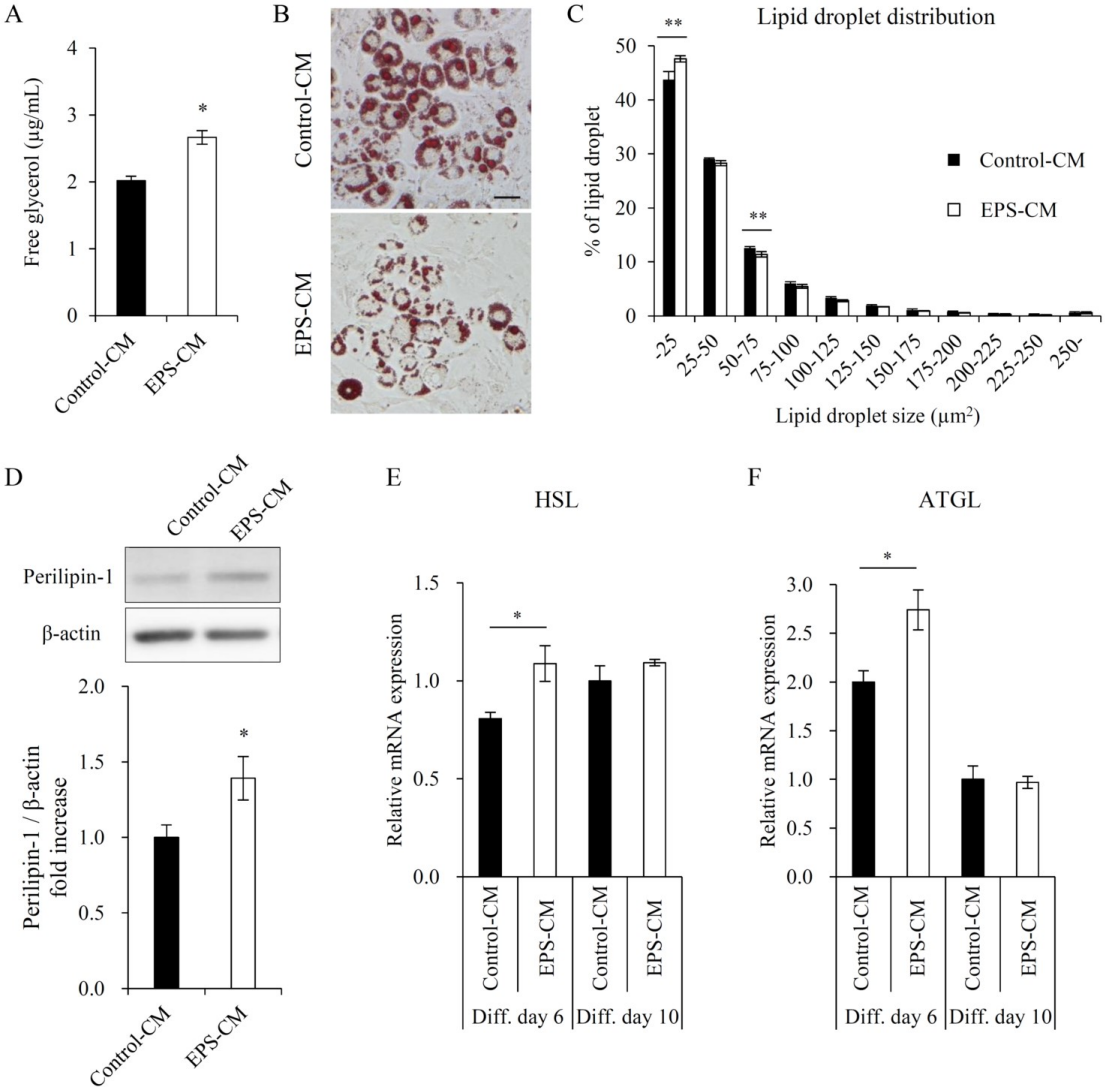
Effects of myotube EPS-CM treatment on lipolysis of 3T3-L1 adipocytes. (A) Following the final 48 h treatment with myotube EPS-CM (days 8–10), glycerol levels in the culture media of 3T3-L1 adipocytes were determined. (B) Representative images of stained mature adipocytes (day 10) containing lipid droplets are shown for the Control-CM and EPS-CM states. (C) The distribution of lipid droplets with respect to their sizes (μm^2^) is shown for the Control-CM and EPS-CM states. (D) Perilipin-1 protein levels in 3T3-L1 adipocytes (day 10) were quantified using immunoblotting. (E, F) The expression of HSL and ATGL was evaluated via qRT-PCR on days 6 and 10. Data are expressed as mean ± SEM (n = 4 (A, C), n = 10 (D), n = 3–4 (E, F)). *; p < 0.05. **; p<0.01. Scale bar, 50 μm.

**Fig 4:**
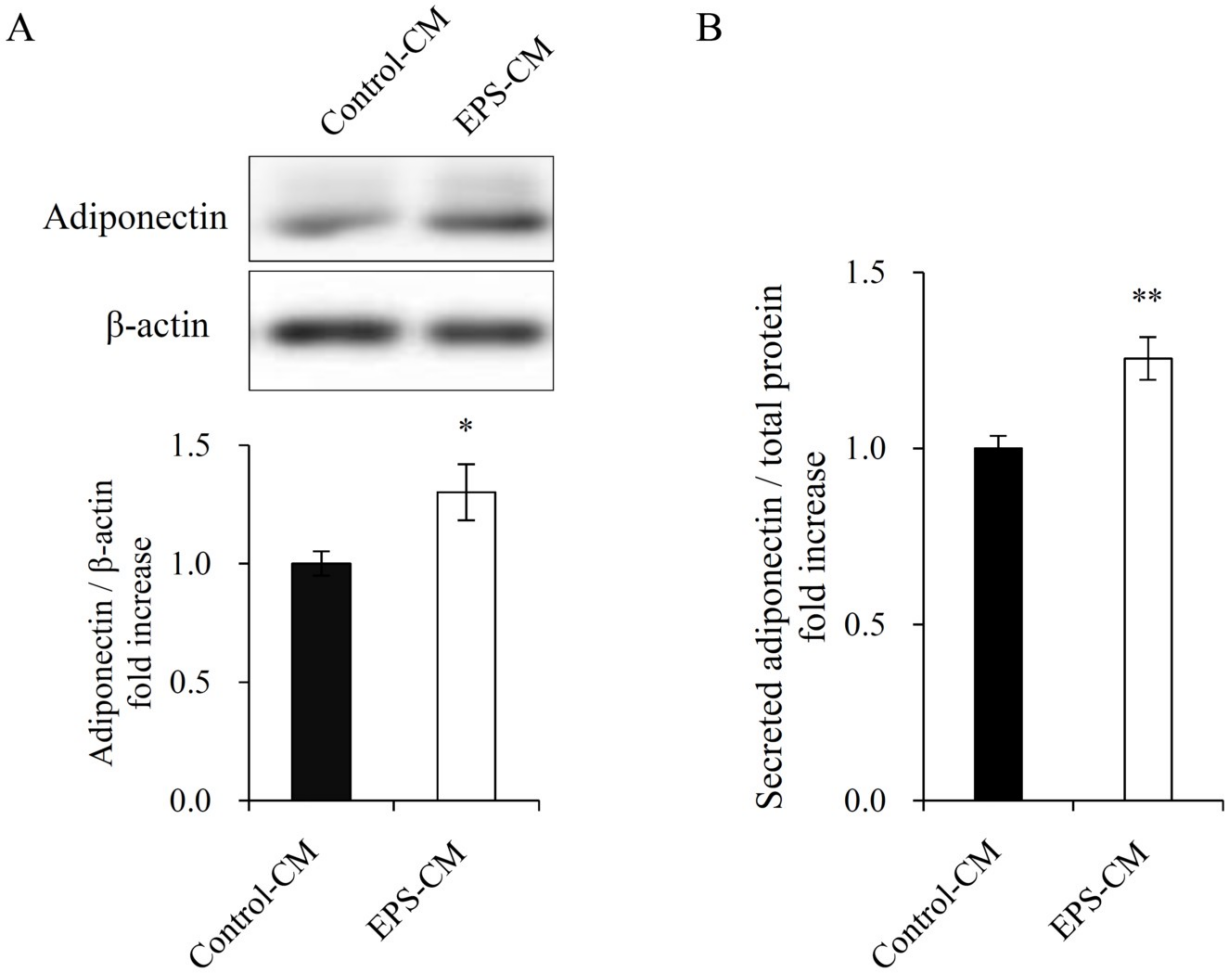
Effects of myotube EPS-CM treatment on adiponectin secretion of 3T3-L1 adipocytes. (A) 3T3-L1 adipocytes were continuously treated with 50% myotube EPS-CM, and on day 10 post-differentiation, cell lysates were subjected to immunoblotting for adiponectin. (B) Following the final 48 h treatment with myotube EPS-CM (day 8–10), the levels of adiponectin in the culture media of 3T3-L1 adipocytes were determined using ELISA. Data are expressed as mean ± SEM (n = 12 (A), n = 8 (B)). *; p < 0.05. **; p<0.01.

**Fig 5:**
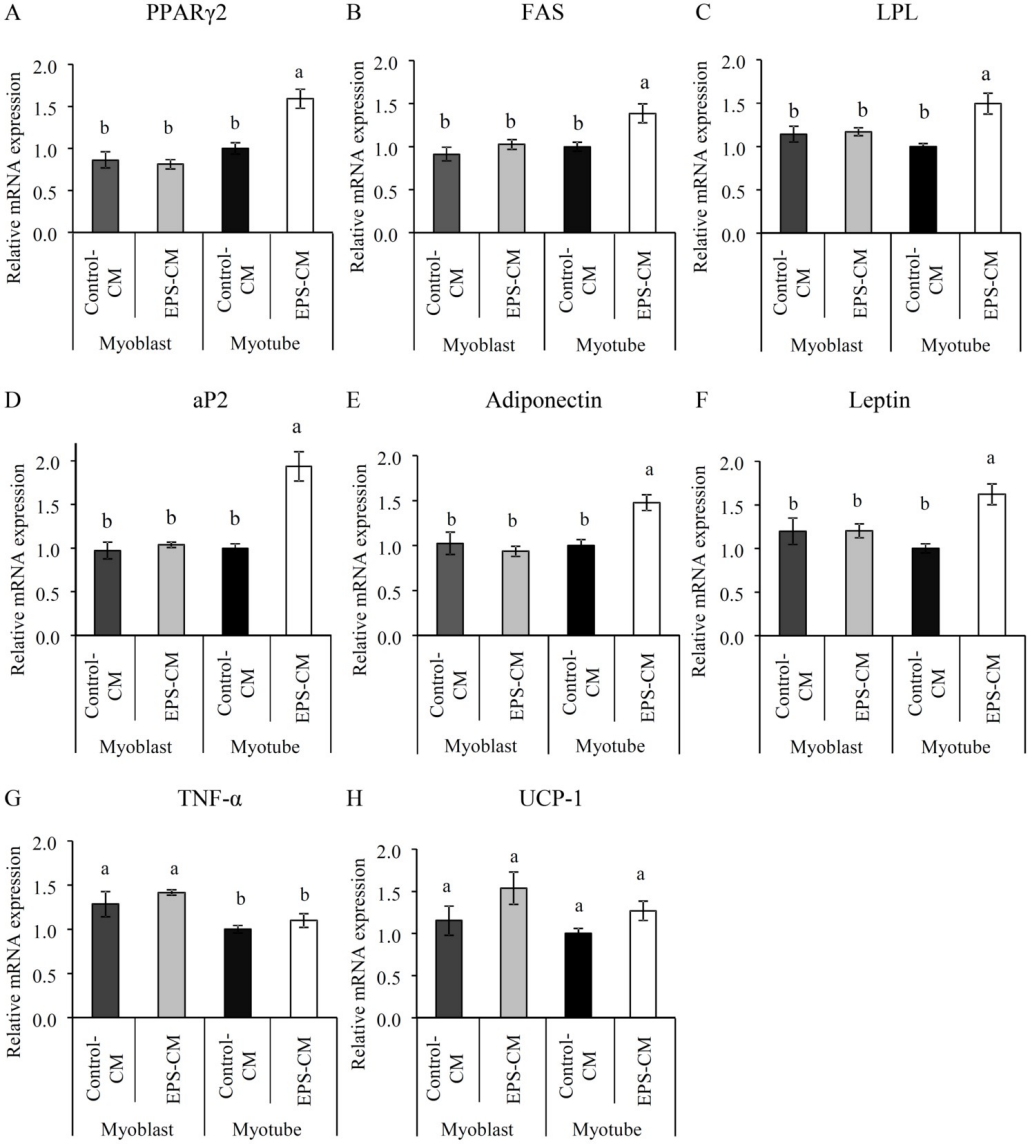
Effects of myotube EPS-CM treatment on mRNA expression of 3T3-L1 adipocytes. The mRNA expression level of 3T3-L1 adipocytes subjected to continuous treatment with C2C12 myotube/myoblast derived CM with and without EPS (EPS-CM and Control-CM) were measured on day 10 post-differentiation. (A-H) The expression levels of PPARγ2, FAS, LPL, aP2, adiponectin, leptin, TNF-α, and UCP-1 were evaluated by qRT-PCR. Data are expressed as mean ± SEM (n = 4–6). Different letters represent significant differences (p < 0.05).

3T3-L1 pre-adipocytes were differentiated via a 1:1 mixture of differentiation medium and C2C12 myotube EPS-CM from day 0 up to a maximum of 10 days. Freshly mixed medium was prepared for each application and changed every 2 d. 3T3-L1 cells treated with EPS-CM were defined as 'EPS-CM,' and those treated with no EPS-CM was defined as 'Control-CM' (Figs 2–5). EPS-CM derived from C2C12 myoblasts was also added to 3T3-L1 differentiation medium, under similar conditions. After treatment with EPS-CM, the cell viability of the 3T3-L1 adipocytes was evaluated using an MTS assay, following the manufacturer's instructions (Cell Titer 96^®^; Promega, Fitchburg, WI), after which the cells were subjected to the following experiments.

### Oil Red O staining and lipids assessment

Four to ten days after treatment with EPS-CM, the 3T3-L1 adipocytes were washed twice with PBS and fixed with 4% paraformaldehyde phosphate buffer solution (Wako, Osaka, Japan) for 20 min at room temperature. The fixed cells were washed three times with PBS and incubated with filtered Oil Red O solution for 20 min. The Oil Red O solution was prepared by mixing 120 mg Oil Red O (Sigma, MO, USA) with 24 mL isopropanol, followed by 16 mL water. After staining, the cells were washed three times with PBS and air-dried at room temperature. Oil Red O was eluted from the stained cells with isopropanol and quantified by measuring absorbance at 510 nm to measure the level of lipid content.

For lipid droplet distribution analysis, bright field images of the stained cells were acquired using an all-in-one fluorescence microscope (BZ-X700, Keyence, Osaka, Japan) at a magnification of 10×. Quantification of lipid droplet size was performed on a hybrid cell counting system (BZ Analyzer; Keyence). Five field images per well were randomly acquired, and the size of each stained droplet was automatically quantified under similar conditions via macro cell count functions. The number of cells containing lipid droplets per field image was also manually counted.

### Lipolysis assay

On day 10 post-differentiation, 48 h following the last EPS-CM treatment, glycerol released in the cell culture media was measured via colorimetry using the free glycerol reagent (Sigma), according to the manufacturer's instructions.

### Immunoblotting

C2C12 myotube extracts were prepared in lysis buffer containing 50 mM Tris-HCl (pH 7.5), 10 mM beta-glycerophosphate, 5 mM sodium pyrophosphate tetrabasic, 1 mM sodium orthovanadate, 1 mM ethylenediaminetetraacetic acid (pH 8.0), 1% Nonidet P-40, 150 mM sodium chloride, 10 mM sodium fluoride, 10 mg/L leupeptin, 3 mM benzamidine, 5 μg/mL aprotinin, and 1 mM phenylmethylsulfonyl fluoride, as previously reported [14]. 3T3-L1 adipocyte extracts were prepared in lysis buffer containing 50 mM Tris-HCl (pH 7.6), 150 mM NaCl, 1% Nonidet P40, 0.5% Sodium Deoxy Cholate, Protease Inhibitor Cocktail, and 0.1% SDS (RIPA Buffer; Nakarai tesque, Kyoto, Japan). Protein concentrations of all samples were measured via a Bradford protein assay (Bio-Rad, Hercules, CA). The lysates or media of C2C12 myotubes and 3T3-L1 adipocytes were separated on an SDS-polyacrylamide gel and transferred onto polyvinylidene fluoride membranes. The membranes were then blocked with Tris-buffered saline, which comprised 0.1% Tween 20 and 5% non-fat dry milk (for adiponectin and β-actin), 5% bovine serum albumin (for perilipin-1), 2.5% non-fat dry milk and bovine serum albumin (for AMPK and phospho-AMPK), and PVDF blocking reagent (TOYOBO, Osaka, Japan; for IL-6 and IL-15). Subsequently, the membranes were incubated overnight with appropriate primary antibodies as follows: anti-adiponectin (A6354, Sigma), anti-perilipin-1 (3470, Cell Signaling Technology, CST, Boston, MA), anti-β-actin (4967, CST), anti-AMPK (2532, CST), anti-phosho-AMPK (2535, CST), anti-IL-6 (AF406NA, R&D Systems, Minneapolis, MN) and anti-IL-15 (MAB447, R&D Systems). For IL-6 and IL-15, the antibodies were diluted using immunoreaction enhancer solution (Can Get Signal; TOYOBO, Osaka, Japan). Rabbit (NA934, GE Healthcare, Piscataway, NJ), goat (sc-2354, Santa Cruz Biotechnology, Dallas, TX) or rat (7077, CST) secondary antibodies conjugated with horseradish peroxidase were used for detection via enhanced chemiluminescence using ECL plus (PerkinElmer Life Sciences, MA, USA). Blots were analyzed using a Luminescent Image Analyzer LAS-4000 mini (GE Healthcare). Data were normalized to background and loading controls.

### ELISA

In order to measure adiponectin in cell culture media, an adiponectin mouse ELISA kit was used according to the manufacturer's instructions (ab108785, Abcam, Cambridge, UK). Concentration was normalized to total protein content.

### RNA extraction and RT-PCR

Total RNA was extracted from 3T3-L1 adipocytes on days 4, 6, and 10 post-differentiation using an RNeasy lipid tissue kit (Qiagen, Hilden, Germany), according to the manufacturer's protocol. RNA was transcribed into cDNA via a standard reverse transcriptase reaction using M-MulV reverse transcriptase (Promega, Madison, WI). Quantitative RT-PCR was performed on a 96-well PikoReal Real-Time PCR System with a DyNAmo ColorFlash SYBR Green qPCR Kit (Thermo Fisher Scientific, MA, USA) or an ABI Prism 7500 with TaqMan Gene Expression assays (Applied Biosystems, Foster City, CA). The mRNA levels of each gene were normalized to those of the housekeeping gene 18S ribosomal RNA (18S rRNA). The primers used in this study were as follows:

PPARγ2, forward: GATGCACTGCCTATGAGCACTT and reverse: AGAGGTCCACAGAGCTGATTCC; FAS, forward: AACTCCAAGTTATTCGACCA and reverse: ACCCAGTTATCTTTCCAGAG; LPL, forward: TATTGGAATCCAGAAACCAG and reverse: GTCAATGAAGAGATGAATGG; aP2, forward: CCGCAGACGACAGGAAGGT and reverse: AGGGCCCCGCCATCT; Adiponectin, forward: GCACTGGCAAGTTCTACTGCAA and reverse: GTAGGTGAAGAGAACGGCCTTGT; Leptin, forward: GGGCTTCACCCCATTCTGA and reverse: TGGCTATCTGCAGCACATTTTG; TNF-α, forward: TCACTGGAGCCTCGAATGTC and reverse: GTGAGGAAGGCTGTGCATTG; UCP-1, forward: CAAGAGTACTTCTCTTCAGG and reverse: CTCTGTAAGCATTGTAGGTC; 18S rRNA, forward: GTAACCCGTTGAACCCCATT and reverse: CCATCCAATCGGTAGTAGCG. For TaqMan Gene Expression assays, C/EBPα: Mm00514283, SREBP-1: Mm00550338_m1, HSL: Mm00495359_m1, ATGL: Mm00503040_m1, 18S rRNA: Mm03928990, respectively.

### Statistical analysis

Statistical analyses were performed using the Ekuseru-Toukei 2012 software (Social Survey Research Information, Tokyo, Japan). Data are expressed as mean ± standard error of the mean (SEM). Differences between two groups were tested using unpaired Student's *t*-tests. Differences among four or more groups were tested via two-way ANOVA followed by Bonferroni *post hoc* test. Statistical significance was set at *p* < 0.05.

## Results

### Preparation of EPS-CM of C2C12 cells

Three-hour contraction induced phosphorylation of AMPK (Thr172) in the myotubes (Fig 1A), whereas LDH activity in the conditioned medium was unaffected by contraction (Fig 1B). These results indicate that the myotubes were not injured by EPS or muscle contraction and did not leak cytoplasmic contents [15, 17]. Lactic acid concentration in the medium increased due to contraction (Fig 1C), while glucose concentration decreased (Fig 1D). We have previously shown that acute contractions by 1 h EPS induced IL-6 and IL-15 secretions from C2C12 myotubes in Krebs Ringer bicarbonate (KRB) buffer [18]. However, the amount of IL-6 and IL-15 in the medium was unchanged under the present EPS protocol (Fig 1E and 1F). Since myoblasts did not contract by EPS (S1 File), the medium collected from myoblasts stimulated by electric pulse was used as a negative control, which can neglect non-contraction related secreted factors.

### Effects of EPS-CM on differentiation of 3T3-L1 pre-adipocytes

First, we investigated the effect of EPS-CM treatment during the differentiation of 3T3-L1 pre-adipocytes. 3T3-L1 pre-adipocytes were incubated with a 1:1 mixture of 3T3-L1 differentiation medium and muscle conditioned medium [19]. We exposed 3T3-L1 pre-adipocytes to myotube EPS-CM for 10 days, and measured the amount of lipids using Oil Red O staining on days 4, 6, and 10, during the course of differentiation (Fig 2A). The amount of lipids was significantly increased by EPS-CM on day 4, and continued to show an increasing trend on day 6, but tended to decrease by day 10 (Fig 2B). We further investigated mRNA expression profiles of key adipogenesis regulators. PPARγ2 expression levels were significantly enhanced by EPS-CM on days 4 and 10 (Fig 2C). C/EBPα and SREBP-1 expression levels remained unchanged at all time points, although C/EBPα expression on day 4 showed an increasing trend (Fig 2D and 2E).

### Effects of EPS-CM on lipolysis of 3T3-L1 adipocytes

Since triglyceride accumulation measured by Oil Red O staining was unchanged but showed a decreasing trend on day 10 when continuously treated with EPS-CM (Fig 2B), we examined whether EPS-CM induced lipolysis in mature adipocytes on day 10. The glycerol level in the culture media of adipocytes treated with EPS-CM was significantly increased from day 8 to 10 (Fig 3A). The size distribution of lipid droplets was also greatly changed by EPS-CM (Fig 3B and 3C). The cell viability and the number of lipid positive cells were unchanged by EPS-CM (S1 Fig). Perilipin-1 is localized on the surface of lipid droplets, and its gene was regulated by PPARγ [20]. Therefore we measured the protein expression level of perilipin-1. As expected, perilipin-1 protein expression was increased on day 10 (Fig 3D), indicating the increased surface area with decreasing lipid droplet size. mRNA expression of hormone-sensitive lipase (HSL) and adipose triglyceride lipase (ATGL), major rate-determining enzymes for lipolysis in adipocytes, were increased by EPS-CM on day 6 but remained unchanged on day 10 (Fig 3E and 3F).

### Effects of EPS-CM on adiponectin secretion of 3T3-L1 adipocytes

Since plasma adiponectin levels are decreased in those with obesity, insulin resistance, or type 2 diabetes [21], adiponectin level is considered to be a biomarker of the metabolic syndrome [22]. We measured adiponectin protein expression in mature adipocytes and its secretion in adipocytes treated with myotube EPS-CM on day 10, and found it to be significantly increased (Fig 4).

### Effects of EPS-CM on mRNA expression of 3T3-L1 adipocytes

To confirm that the changes in mature adipocytes were induced by myotube EPS-CM (Figs 1–4), we quantified mRNA expression levels of differentiation markers on day 10. The mRNA expression levels of PPARγ2 and PPARγ-regulated genes such as fatty acid synthase (FAS), lipoprotein lipase (LPL), and apolipoprotein 2 (aP2) were significantly increased in 3T3-L1 adipocytes treated with myotube EPS-CM (Fig 5A–5D). Since the upregulation of PPARγ is believed to induce secretion of adipokine, adiponectin, and leptin, we next examined the expression levels of these genes. As expected, the expression levels of these genes were significantly upregulated by myotube EPS-CM (Fig 5E and 5F). We confirmed that the gene expression levels in 3T3-L1 adipocytes treated with myoblast EPS-CM were not changed (Fig 5A–5F). On the other hand, the mRNA expression levels of uncoupling protein (UCP)-1 and tumor necrosis factor-alpha (TNF-α) were unchanged by myotube EPS-CM (Fig 5G and 5H).

## Discussion

The objective of the current study was to identify a novel muscle–adipose tissue crosstalk process. We used a C2C12 myotube contraction system controlled by EPS and treated 3T3-L1 adipocytes with the myotube EPS-CM. Our results demonstrated that continuous treatment with myotube EPS-CM promoted lipid accumulation of 3T3-L1 pre-adipocytes (Diff. day 4) (Fig 2), via the upregulation of PPARγ2 (Fig 2C). Furthermore, myotube EPS-CM promoted lipolysis (Fig 3), secretion of adiponectin (Fig 4), and expression of PPARγ-regulated gene expression (Fig 5) in mature adipocytes (Diff. day 10). To our knowledge, this is the first report indicating that myokines promote PPARγ2 or adiponectin expression in adipocytes.

A previous study showed that lactic acid at non-physiological concentrations (10 mM) enhanced lipid accumulation by inducing adipogenesis [23]. Although, in our study, the lactic acid level in the myotube culture media was increased by EPS (Fig 1C), the level was below normal physiological concentrations and too low to affect 3T3-L1 adipocytes. High glucose also promotes adipogenesis compared to low glucose (25 mM vs. 4 mM) [24]. EPS significantly reduced the glucose concentration in the myotube culture media via contraction-induced glucose consumption, but to a very small degree (Fig 1D); the estimated glucose concentration in basal and contraction was 15 mM and 14.85 mM, respectively, after treatment with 1:1 mixture of media. Therefore, lactic acid or glucose in EPS-CM is unlikely to affect lipid accumulation in the present study. The secretion of IL-6 and IL-15 was unchanged under the present EPS protocols (Fig 1E and 1F). The difference may depend on the medium, as KRB buffer, rather than DMEM, is suitable for detecting IL-6 secretion [18]. Regardless, it is unlikely that these proteins affected adipogenesis or lipolysis in the present experiment system, even though it has been reported that IL-6 and IL-15 reduce adiposity [6–9]. Other myokines, Irisin and Meteorin-like, which are secreted by cultured muscle cells overexpressing PGC1-α, promote adipose tissue browning [10, 11]. However, these myokines do not exert an effect on PPARγ or adiponectin mRNA expression in adipocytes [10, 25]. However, we did not confirm the levels of these myokines in EPS-CM. It is still possible that musclin, which is an activity-stimulated myokine [26], has the potential to reduce adiposity [27], and its secretion by EPS or the effect on adipocytes should be examined in future studies. Therefore, enhanced PPARγ pathway in 3T3-L1 adipocytes by myotube EPS-CM may be mediated by unidentified molecule(s) secreted by contraction.

PPARγ activates the transcription of adipocyte-related genes that promote adipogenesis and is the molecular target of anti-diabetic drugs, thiazolidinediones [28]. The increased lipid accumulation observed in differentiating 3T3-L1 pre-adipocytes following myotube EPS-CM treatment (Fig 2A and 2B) is similar to the effect of anti-diabetic drugs *in vitro* [29]. These drugs are considered to exert their anti-diabetic action by inducing the expression of PPARγ-regulated genes involved in lipid and glucose metabolism, such as adiponectin [28]. While these drugs act as PPARγ ligands, the present study confirmed that EPS-CM rather increased the expression of PPARγ2 itself, both in pre-adipocytes and mature adipocytes (Figs 2C and 5A), and subsequently increased the expression of PPARγ-regulated genes (Fig 5B–5F). C/EBPα is also a critical transcription factor in adipogenesis. It has been reported that C/EBPα and PPARγ positively regulate each other's expression [30]. C/EBPα expression showed an increasing trend by EPS-CM on only day 4 (Fig 2D). This is in line with a previous study, which reported that C/EBPα plays important roles in the early phase of pre-adipocyte differentiation [30]. However, C/EBPα is not essential for adipogenesis, and PPARγ has a greater contribution to adipogenesis [31]. We speculate that the changes in adipocytes in this study could be explained by increased PPARγ2 expression. Since the regulator of PPARγ expression is also reported to represent another approach to the treatment of insulin resistance [29], unidentified molecule(s) secreted in EPS-CM sheds light on the possible mechanism by which exercise prevents obesity and type 2 diabetes.

On day 10 of differentiation, continuous treatment with myotube EPS-CM induced glycerol release and reduced lipid droplet size in mature adipocytes (Fig 3A–3C), although triglyceride accumulation and efficacy of differentiation remained unchanged (Fig 2B and S1 Fig). These findings suggest that the formation of lipid droplets with decreasing size reflects the increased surface area, which may be consistent with the increased perilipin-1 expression (Fig 3D). Lipid droplet formation in mature adipocytes is regulated by enzymes that predominantly regulate the hydrolysis of triglycerides, HSL and ATGL. Since activation of HSL is regulated by phosphorylation modulated by cAMP-dependent PKA, changes in HSL expression have a limited impact on lipid droplet size [32]. On the other hand, changes in ATGL expression are critical for the hydrolysis of triglycerides in the absence of PKA-dependent adrenergic stimulation (basal lipolysis) [33], and influence lipid droplet size, regardless of perilipin-1 activity [32]. The current study indicated that continuous treatment with myotube EPS-CM increased the expression of HSL and ATGL on Diff. day 6, where the increase in ATGL expression was greater than that of HSL expression (Fig 3E and 3F). Although neither HSL nor ATGL expression changed on Diff. day 10 (Fig 3E and 3F), ATGL expression may have increased due to EPS-CM treatment during late stages of differentiation (Diff. days 6–10). EPS-CM treatment may have increased ATGL expression, resulting in increased lipolysis and decreased lipid droplet size. Although ATGL expression is PPARγ-dependent [34], the response to EPS-CM in the present study was varied. A possible cause may be AMPK activation, as ATGL expression and basal lipolysis are reportedly regulated in an AMPK-dependent manner [35]. Although the regulation of basal lipolysis should be investigated in detail in future studies, these findings suggest that a novel communication from muscle to mature adipocytes directly induced lipolysis in a sympathetically independent manner. This could explain the exercise-induced reduction of intermuscular fat [36].

Adiponectin is an insulin-sensitizing, anti-inflammatory, and anti-atherogenic hormone. On Diff. day 10, continuous treatment with myotube EPS-CM increased both adiponectin expression and secretion (Figs 4 and 5E). The results are consistent with those of a previous finding that decreasing fat cell size was associated with adiponectin synthesis and secretion [37]. Furthermore, the results may support a previous study indicating that short-term exercise upregulates circulating adiponectin levels without body composition changes [38]. Unidentified myokines secreted by muscle contraction may directly enhance adiponectin production in adipose tissue. On the whole, treatment with myotube EPS-CM promoted adipogenesis in every phase of differentiation, followed by enhanced adiponectin secretion, while simultaneously affecting lipolysis and lipid droplet formation in mature adipocytes. This discrepancy suggests that the alteration of PPARγ expression was not the sole factor. We speculate that there might be other mechanisms contributing to this phenomenon.

Evers-van Gogh et al. suggested that metal ions (i.e., Zn and Cu) generated in the culture medium by EPS could unexpectedly affect recipient cells (hepatocytes in that study) [19]. The current study controlled non-cell-mediated effects by focusing on the effects of myotube EPS-CM rather than myoblast EPS-CM (Fig 5). Myoblasts, which are in an undifferentiated state, do not contract when subjected to EPS (S1 File). Additionally, myoblast EPS-CM tested in the present study did not contain contraction-induced myokines. Therefore, we conclude that PPARγ2 and PPARγ-related gene expressions were induced by contraction-induced myokines, especially from myotubes.

## Conclusions

In conclusion, we demonstrated that myotube EPS-CM induced PPARγ2 and PPARγ-regulated gene expression in adipocytes and thereby promoted adipogenesis and lipid metabolism. We showed that our experimental system, in which myotube EPS-CM is added to recipient cells, is a useful *in vitro* model for investigating the physiological functions of myokines, directly acting on distant organs. We expect to identify the factors responsible for this phenomenon in a future study.

## Supporting information

S1 File Contractile movement of myotubes and myoblasts evoked by EPS. C2C12 myotubes contracted on stimulation with electric pulses of 35 V, 15–20 mA at 1 Hz for 25 ms at 975-ms intervals, while myoblasts did not. The movie was acquired using a phase-contrast microscope (Biozero, Keyence) at a magnification of 20 ×.
(WMV)

S1 Fig (A) 3T3-L1 adipocytes were continuously treated with 50% myotube EPS-CM, and on day 10 post-differentiation, the media were subjected to MTS assay for evaluating cell viability. (B) The number of lipid positive cells per field image is shown for the EPS-CM and Control-CM states (day 10). Data are expressed as mean ± SEM (n = 4). N.S., not significant.
(TIFF)

S2 Fig Raw images of immunoblotting. (PDF)
