# Supplementary figures and images for: Effect of treatment with conditioned media derived from C2C12 myotube on adipogenesis and lipolysis in 3T3-L1 adipocytes
Source: PLoS One. 2020 Aug 5;15(8):e0237095. doi: 10.1371/journal.pone.0237095 (PMC7406041; doi:10.1371/journal.pone.0237095)

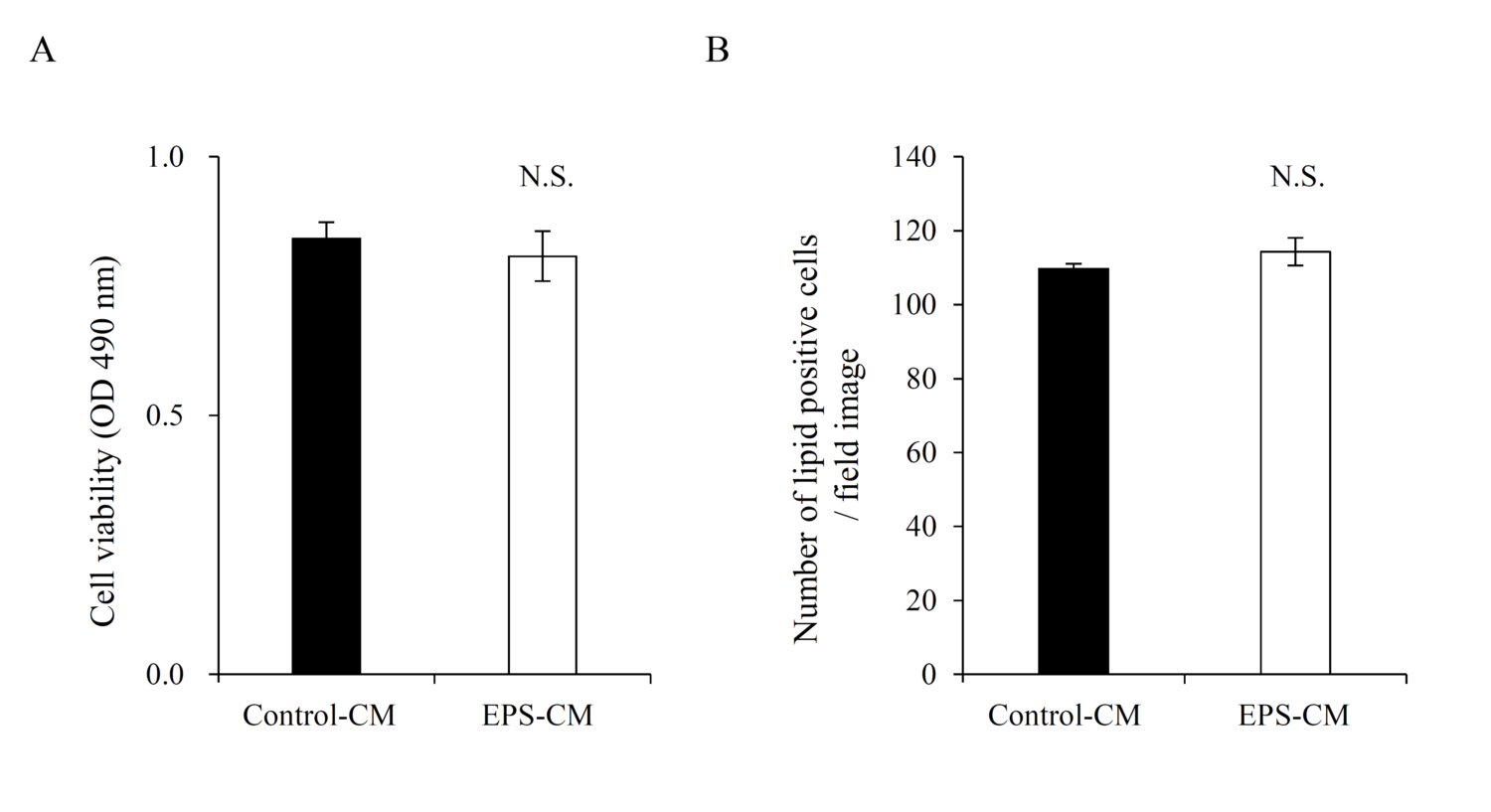

Supplement: S1 Fig — (A) 3T3-L1 adipocytes were continuously treated with 50% myotube EPS-CM, and on day 10 post-differentiation, the media were subjected to MTS assay for evaluating cell viability. (B) The number of lipid positive cells per field image is shown for the EPS-CM and Control-CM states (day 10). Data are expressed as mean ± SEM (n = 4). N.S., not significant. (TIFF) [file pone.0237095.s002.tiff]
