# Supplementary material for: Effect of treatment with conditioned media derived from C2C12 myotube on adipogenesis and lipolysis in 3T3-L1 adipocytes
Source: PLoS One. 2020 Aug 5;15(8):e0237095. doi: 10.1371/journal.pone.0237095 (PMC7406041; doi:10.1371/journal.pone.0237095)

Blot images of Fig 1A, E, F

A

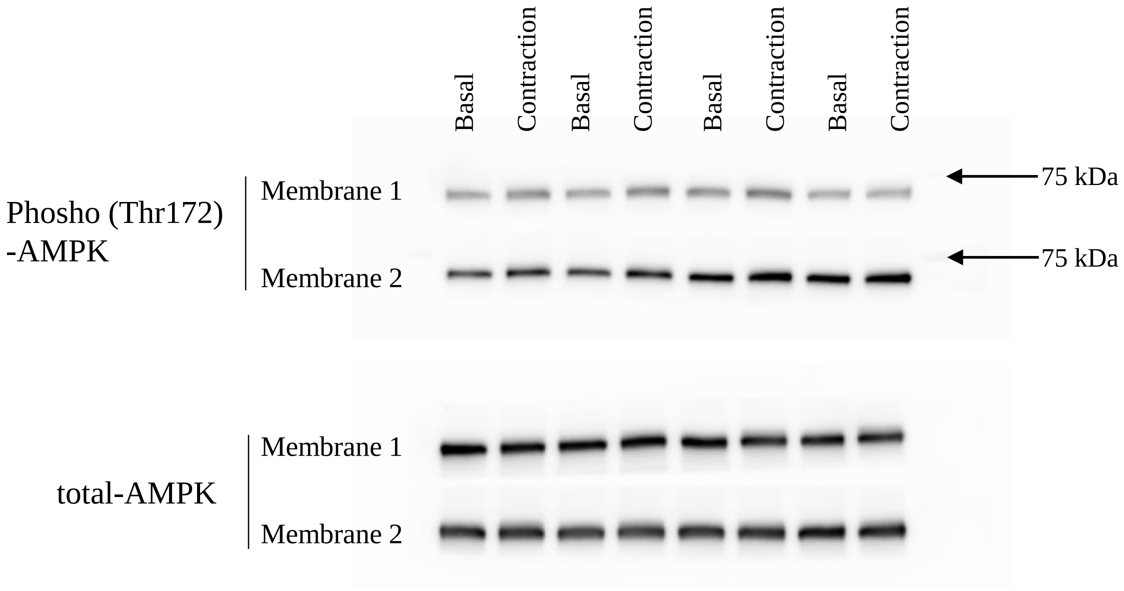

E

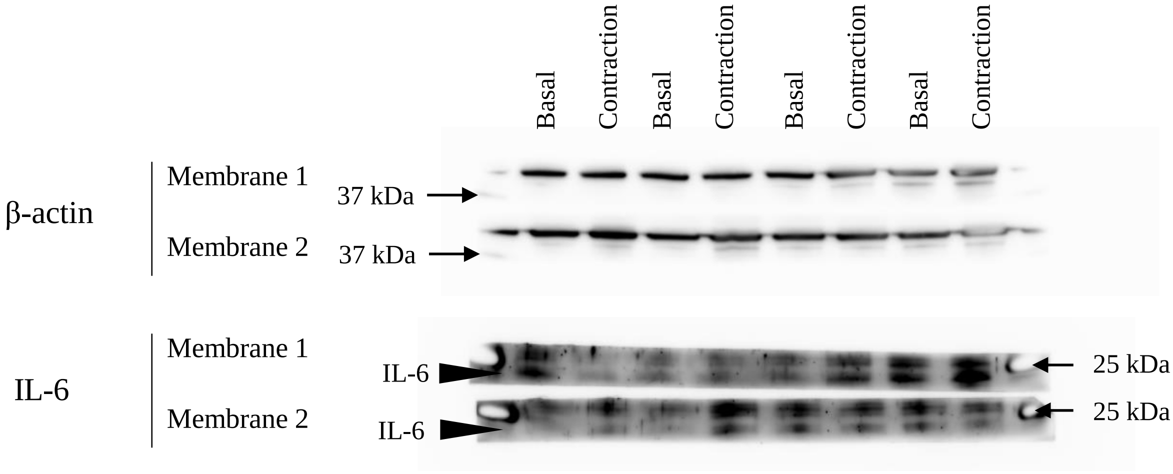

F

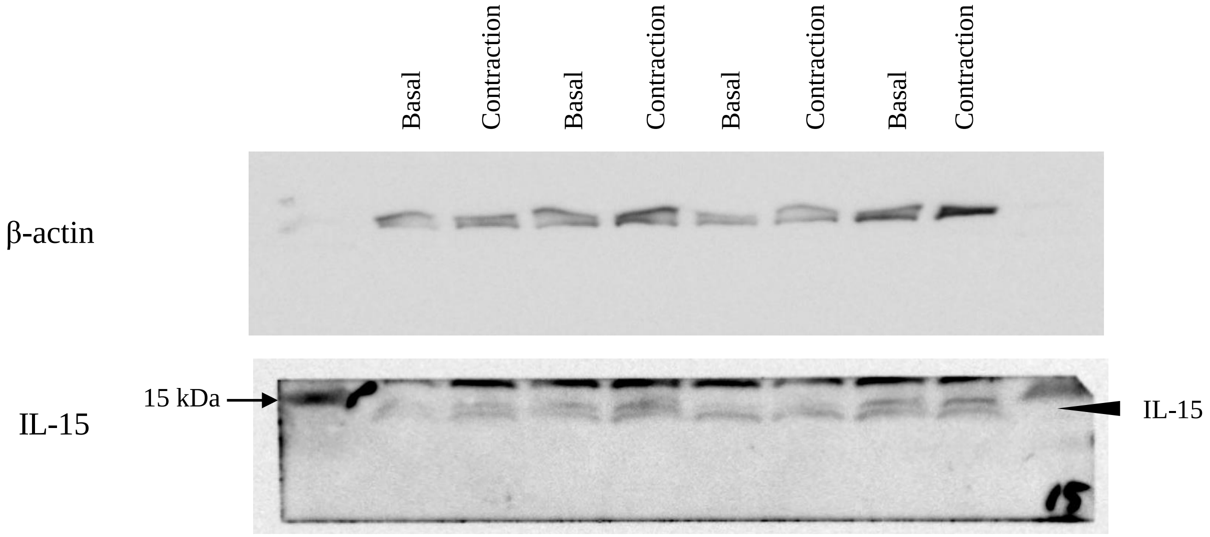

Detection of IL-6 and IL-15 in the myotube EPS-CM was validated in our previous study [17].

Blot images of Fig 3D & 4A (1)

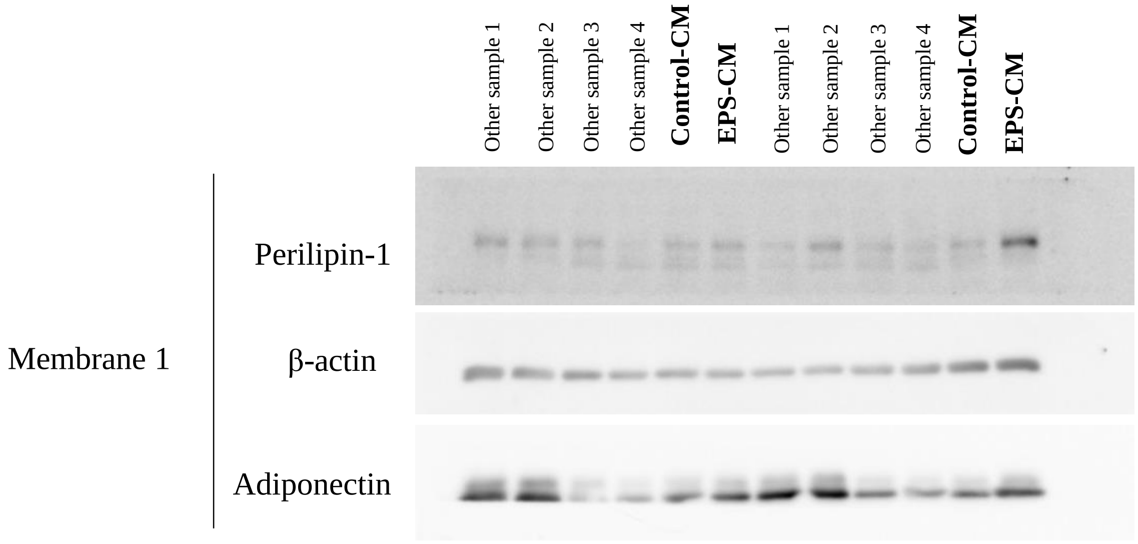

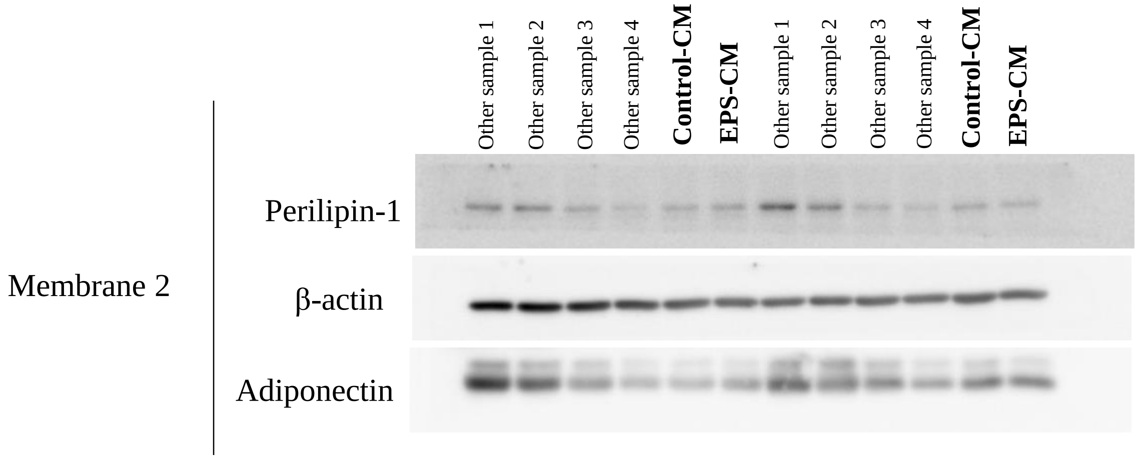

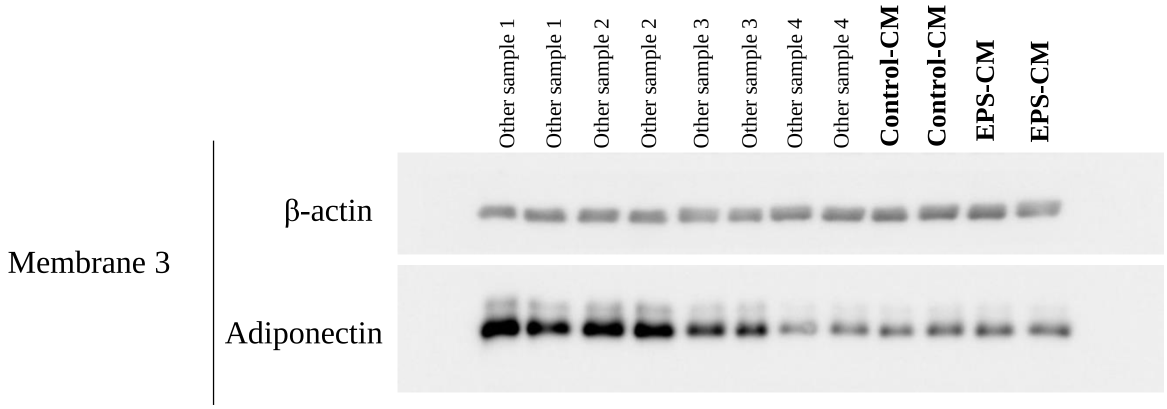

Membranes were cut into three pieces and perilipin-1 (62 kDa), β-actin (45 kDa), and adiponectin (27 kDa) were detected, respectively. Other samples were excluded from the results.

Other sample 1, 2: non-cell Control-CM, EPS-CM.

Other sample 3, 4: myoblast Control-CM, EPS-CM.

Other sample 5, 6: <3 kDa fraction of myotube Control-CM, EPS-CM.

Other sample 7, 8: ≥3 kDa fraction of myotube Control-CM, EPS-CM

Blot images of Fig 3D & 4A (2)

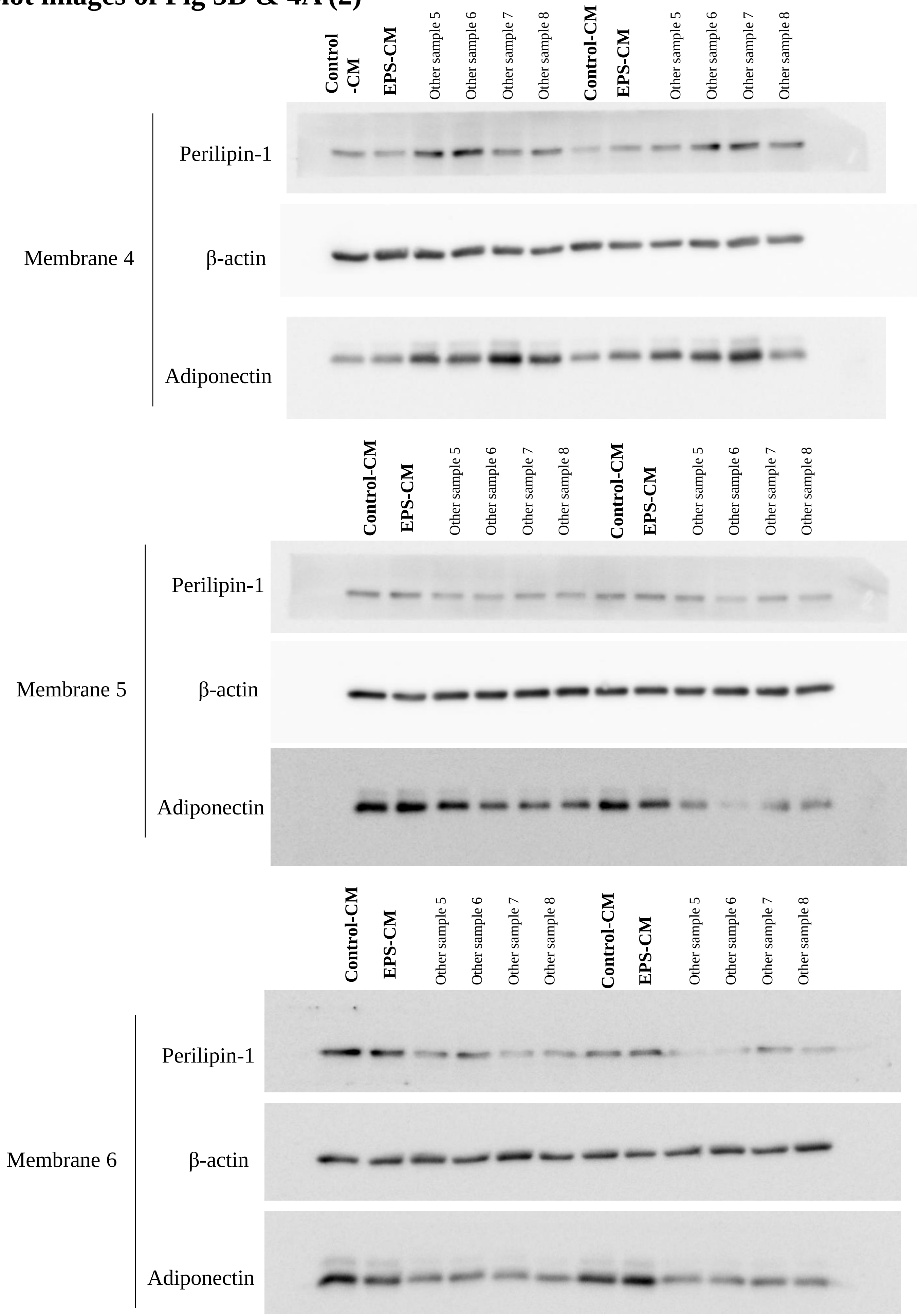

Supplement: S2 Fig — (PDF) [file pone.0237095.s003.pdf]
